# Effect of L- Arginine On Electrocardiographic Changes Induced By Hypercholesterolemia And Isoproterenol In Rabbits

**Published:** 2009-01-07

**Authors:** Pradeep Kumar, Manish Goyal, J L Agarwal

**Affiliations:** 1 Assistant Professor, Deptt of Physiology, CSM Medical University, Lucknow 226003. (KG Medical University); 2 Senior Resident, Deptt of Physiology, All India Institute of Medical Sciences New Delhi 110011; 3 Professor, Department of Physiology, Govt Medical College, Dharmshala at Tanda, Himachal Predesh

**Keywords:** L-arginine, QTc, hypercholesterolemia, rabbit

## Abstract

Hypercholesterolemia, a well-known cardiovascular risk factor, is associated with prolonged action potential duration, longer QTc intervals (rate controlled QT interval), suggested that Hypercholesterolemia may have a direct effect on ventricular repolarization. Hypercholesterolemia was induced in rabbits and L-arginine was given orally to animals for sixteen weeks. The isoproterenol was injected in all the animals to produce electrocardiographic changes. ECG was recorded in lead II at start of study, after hypercholesterolemic diet and/ or L-arginine supplementation.

It is observed that L-arginine significantly reduced the hypercholesterolemia induced QTc prolongation. Isoproterenol induced increase in QTc intervals were decreased only in normolipidemic animals. No significant changes were observed in QRS complex and heart rate. Our study suggests that L-arginine definitely have effect on repolarization processes of myocardium.

## Introduction

Long QT Syndrome is a disorder characterized by delayed cardiac repolarization and increased risk of developing potentially fatal ventricular arrhythmias. The delayed rectifier current (IK) is a major determinant of the phase 3 of the cardiac action potential. It comprises two independent components: one rapid (IKr) and one slow, (IKs). Slowly activating potassium channel are responsible for prolongation of QT. The activity of some K+ channels is drastically altered by the oxidation of critical SH-groups of the channel protein [1-4].

Hypercholesterolemia is a well-known cardiovascular risk factor, and it can induce myocardial electrical remodeling and increases vulnerability to ventricular fibrillation, which was associated with prolonged action potential duration, longer QTc intervals (rate controlled QT interval) and increased repolarization dispersion, suggested that hyperlipidemia may have a direct effect on ventricular repolarization [5-7]. Previous studies have also been reported that morbid obesity is associated with prolongation of QTc interval [8,9]. Diet-induced hypercholesterolemia in rabbits caused contractile dysfunction independent of vascular disease, characterized by a decrease in the maximum rate of shortening and relaxation in a papillary muscle preparation [10].

Nitric oxide (NO), essential for the proper functioning of the cardiovascular system, is derived from L-arginine by NO synthase (NOS) in endothelial cells. NO synthase inhibition produces various cardiovascular abnormalities and ventricular contractile dysfunction [11-15]. NO donors or the precursor for NO synthesis, L-arginine, can ameliorate reperfusion-induced arrhythmias and reduce ischemic/ reperfusion injury in rabbits [16]. Increased activity of myocardial NOS and NO attenuates beta adrenergic responsiveness and over production of NO provide resistance against epinephrine induced arrhythmia. Hyperglycemia induced prolongation of QT values in isolated hearts were prevented almost completely by L-arginine [18].

The role of L-arginine in hypercholesterolemia induced long QT interval and repolarization related abnormality is yet to be endeavored. Therefore in present study we evaluated the effect of oral administration of L-arginine on hypercholesterolemia as well as isoproterenol induced arrhythmic changes in rabbits.

## Material and Methods

Present study approved by institutional ethics committee was conducted on 24 adult male albino rabbits (Indian breed) between 9-10 months old and weighing 1.2 to 1.4 kg body wt. The animals were employed in the study were kept as described earlier [19], briefly animals were acclimatized in ambient conditions of animal house and all experimental procedures were performed in accordance with institutional animal care guidelines. Each animal was fed with 120 gm standard rabbit feed (SRF) (Amrut, Maharashtra) per day in divided doses and water intake was maintained ad libitum. Animals were randomly divided in four groups.

Group-1 (Control): Animals (n=6) of this group kept on standard rabbit feed (SRF) 120 gm per day were recognized as control.

Group-2: Animals (n=6) of this group provided with standard rabbit feed and L-arginine (100/mg kg body wt orally).

Group -3: Hypercholesterolemic diet along with standard rabbit feed was given to animals (n=6) of this group.

Group- 4: L-arginine 100 mg/kg body wt given orally along with Hypercholesterolemic diet and standard rabbit feed to animals (n=6) of this groups.

### Protocol - I : Intervention of Diet and L-Arginine

#### Hypercholesterolemic diet

Animals of group-3 and group-4 made hypercholesterolemic by method employed earlier Kumar et al. [20], briefly the animals were kept on diet containing 0.2% cholesterol and 3% coconut oil enriched with 120 gm standard rabbit feed daily for sixteen weeks, L-arginine 100mg/kg body wt orally were given to animals of group-2 and group-4 and water intake was maintain ad libitum. Serum cholesterol of all the animals was estimated using kit from Span diagnostics India to assess the hypercholesterolemia.

#### Collection of blood sample

The fasting venous blood samples were taken from the ear vein of rabbits using scalp vein set 24G. 1.0 ml blood was collected in 2.0 ml glass syringe and the serum was finally separated out by centrifugation.

#### Biochemical estimation

Serum total cholesterol was estimated by using principle of Wybenga and Plegg [21] and the reagent kit procured from Span Diagnostics India.

### Recording of electrocardiogram of rabbits

ECG of all the conscious animals was recorded by securing the rabbit on wooden table in temperature controlled (25º-28ºC) room; applying special copper disc electrodes (10 mm diameter) and ECG machine (CHARDIART 508 BPL India). ECG at start of study, after protocol-I and after protocol-II was recorded in lead-II for 3 minutes.

### Protocol – II: Induction of ECG Changes

Experimental QTc changes were induced in animals of all the groups giving single subcutaneous injection of isoproterenol (0.20 mg/kg, i.p.) [22]. The ECGs were recorded 5 minutes after the injection to assess the effect of isoproterenol on heart rate, QRS complex and QT interval. The QTc (rate controlled QT interval) were calculated.

### Electrocardiographic analysis

The ECG recordings were taken with a paper speed of 50 mm/sec at normal filtering. ECG parameters were measured manually; QRS duration was defined as the maximum QRS duration in any lead from the first to the final sharp vector crossing the isoelectric line. QT interval was measured from the lead II using calipers. QT interval was defined as the interval between the beginning of QRS complex and the end of T wave. The onset and offset of T wave were defined as the intersections of the isoelectric line and the tangent of the maximal slope on the up and down limbs of T wave, respectively. Three consecutive cycles were measured in each of standard lead-II, and a mean value was calculated from the three values. Rate-corrected QT values (QTc) were derived using the formula (Mitchell et al., 1998) QTc = QT/SQRT (RR/100) and these were represented as QTc. [23]

### Statistical analysis

All data are expressed as the mean ± SD; differences between groups were calculated using SPSS-13 software. The treatment and control group were compared by non parametric analysis.

## Results

### Changes in cholesterol levels after protocol- I

Baseline characteristics and the changes in cholesterol levels after hypercholesterolemic diet and/or L-arginine therapy of all the animals in this study are shown in Table 1. At baseline, total cholesterol levels were 78.60 ±2.50 mg/dl .No significant changes in cholesterol levels were observed after 16th weeks the in control (group-I). It is observed that in Group-2 the cholesterol level raises >34%, while in Group-3 and Group-4 this increase was >230% and >279% respectively (p<0.001). Above data revealed that hypercholesterolemic diet successfully induces the hypercholesterolemia. It is also observed that in L-arginine supplemented animals (Group-2 and Group-4) the cholesterol levels were raises significantly greater than that of without L-arginine fed animals (Group-1 and Group-3).

### Changes in electrophysiological parameters after protocol- I

After protocol- I no significant change either in the heart rate or QRS complexes were observed, while significant changes in QTc were seen in animals supplemented with hypercholesterolemic diet and/or L-arginine for sixteen weeks (Table 2a and 2b). QTc was increases significantly in (hyperlipidemic diet fed animals) Group-3 (p<0.01) and Group-4 (p<0.05). But increase in QTc was significantly lesser (p<0.05) in Group-4 as compared to group-3.

### Changes in electrophysiological parameters after protocol- II

No mortality were observed during whole of the study, significant changes were seen in almost all the ECG parameters and in all the groups after injection of isoproteronol. Significant increase in heart rate ST elevation and T inversion observed in all the groups, while no significant (p>0.05) changes were observed in QRS complexes in various groups after isoproteronol injection (after protocol-II). QTc intervals were increased significantly (<0.05) in all the groups including control. Increase in QTc was not significant in group-4 and group-2 as compared to pre isoproterenol state. The increase in QTc was significantly (<0.05) lesser in L-arginine fed animals. (Group-2 and group-4) (Table 2a and 2b)

## Discussion

Present study has been planned to evaluate the effect of L-Arginine supplementation on basal electrocardiographic parameters and after isoproterenol induced ischemia in normocholesterolemic and hypercholesterolemic rabbit model. Hypercholesterolemia par se is known to causes repolarization abnormalities probably by beta mediated or IK channel phosphorylation mediated mechanism. Various studies with NO donor, NO synthase inhibitor shown that nitric oxide has potential role in cardio protection as NO attenuates the beta mediated effects of repolarization characteristics [11-18]. Therefore NO may be considered as physiological anatagonist for hypercholesterolemic induced risk of repolarization abnormalities. L-Arginine, a NO precursor, has not been tried in reduction of prolonged QTc in experimental hypercholesterolemic animals.

In present study hypercholesterolemic state in rabbits has been successfully induced by feeding them hypercholesterolemic diet over a period of 16 weeks. L-Arginine supplementation also resulted in a little (> 34%) but significant increase in cholesterol levels. The same effects on serum cholesterol levels have been reported earlier but mechanism underlying this change is not yet known. [20]

Corrected QT interval (QTc) as a marker for repolarization characteristics was significantly increased in cholesterol fed animals with or without L-Arginine supplementation. Hypercholesterolemia itself increases risk of heart diseases and prolongation of QTc interval by altering contractile property of myocardium [10]. Previous studies in animals as well as in humans has been reported a prolongation of QTc in hypercholesterolemic states and is considered to be due to increased oxidative stress and myocardial remodeling [6,7]. The increase in QTc interval was lesser in L-Arginine supplemented hypercholesterolemic group, suggesting the preventive and beneficial role of L-Arginine in hypercholesterolemia induced repolarization characteristics.

Isoproteronol administration leads to global ischemia and arrhythmia in-vivo [22]. During the protocol no mortality was observed. Ischemic changes like ST elevation and T inversion were seen in all the groups after injection of isoproteronol. The isoproteronolol challenge induced arrhythmia risk (Prolongation of QTc interval) was decreased significantly in L-Arginine supplemented hypercholesterolemic animals.

This reduction in risk may be attributed to the fact that NO attenuates beta mediated autonomic modulation of repolarization abnormalities. The reduction in the risk of arrhythmia after ischemic challenge in L-Arginine supplemented group, further strengthen the cardio-protective role of NO.

QTc interval represented the repolarization of myocardium and K+ channels set the membrane potential as well as the excitability of most living cells. The K+ ions are predominantly responsible for the long QTc. It is obvious that L-Arginine increases endogenous NO and an increase in nitric oxide activity may activate ATP dependent K+ channel, which plays a major role in the shortening of action potential [24]. Isoproterenol is beta adrenergic agonist hence alter the K+ conductance and repolarization duration [4]. It seems reasonable that the reduction in isoproteronol induced prolongation of QTc in normolipidemic groups and hypercholesterolemia induced prolongation of QTc in hyperlipedemic groups by L-Arginine in our study is may be due to change in K+ conductance in myocardium.

Nitric oxide acts centrally and enhances parasympathetic tone which in turn secreted acetylcholine (ACh) in its nerve endings [14]. ACh in myocardium alter the K+ conductance and repolarization of myocardium. No significant change in basal heart rate in L-Arginine supplemented animals of our study suggest that centrally acting nitric oxide may not be involved in the alteration of QTc intervals in our study.

The exact mechanism responsible for reduction in prolonged QTc in hypercholesterolemic animals in this study is not known. But it can be speculated that oral L-Arginine increases endogenous NO and in turn alter the activity of ATP dependent K+ channel hence repolarization. QTc prolongation is reported to be associated with malignant arrythmias and sudden cardiac death [7-9]. Therefore, a reduction in QTc prolongation may lead to a reduction in the rate of arrythmias and sudden cardiac death.

## Conclusion

Oral administration of L-Arginine reduces hypercholesterolemia induced and isoproteronolol induced QTc prolongation. The beneficial role of L-Arginine needs to be evaluated for clinical human studies.

## Figures and Tables

**Table 1:**
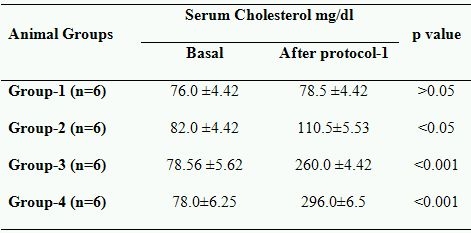
Effects of L-Arginine and hyperlipedemic diet

**Table 2a:**
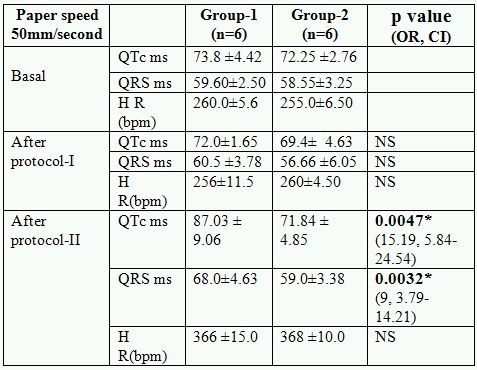
Effect of L-arginine on electrocardiographic parameters in normolipidemics

* Significant p value (OR: odd ratio, CI: confidence interval, NS: not significant)

**Table 2b:**
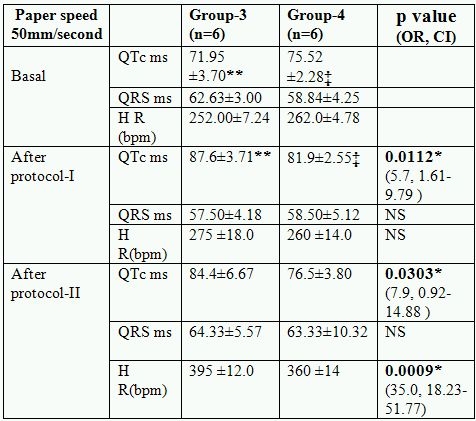
Effect of L-arginine on electrocardiographic parameters in hyperlipidemics

* Significant p value(OR: odd ratio, CI: confidence interval, NS: not significant); ‡ p<0.001 (QTc basal vs QTc after protocol-I in group-4); * * p <0.0001 QTc basal vs QTc after protocol-I and QTc after protocol-I vs protocol-II in group-3
